# Drill irrigation during in vitro drilling with three static computer-assisted implant surgery systems

**DOI:** 10.4317/medoral.27560

**Published:** 2026-01-24

**Authors:** Tania Moya-Martínez, Adrià Jorba-García, Sebastián Lobos-Grimaldi, Iván Valdés-Berritzbeitia, Javier Bara-Casaus, Rui Figueiredo, Eduard Valmaseda-Castellón

**Affiliations:** 1 Oral Surgery and Implantology, Faculty of Medicine and Health Sciences, University of Barcelona, Barcelona, Spain; 2 Oral and Maxillofacial Surgery, Mutua Terrassa University Hospital, Terrassa,Spain; 3 IDIBELL Institute, Barcelona, Spain; 4 Oral Rehabilitation and Maxillofacial Prostheses, Faculty of Medicine and Health Sciences, University of Barcelona, Barcelona, Spain; 5 Dental and Maxillofacial Institute, Sagrat Cor University Hospital, Barcelona, Spain; 6 Oral Surgery, Faculty of Medicine and Health Sciences, University of Barcelona, Spain

## Abstract

**Background:**

Static computer-assisted implant surgery (sCAIS) allows for accurate implant placement. However, the currently available systems usually block drill irrigation, increasing the risk of overheating and tissue damage. The present in vitro study evaluates the volume of irrigation solution that passes through the guide sleeve during implant drilling with different sCAIS systems.

**Material and Methods:**

The volume of irrigation solution that passed through a designed guide sleeve was measured on a 5ml syringe. The following sCAIS systems and groups were evaluated: A sleeve-in-sleeve with drill handle system (Straumann® sCAIS system); an integrated sleeve-in-drill system (RealGuide Z3D sCAIS system), and an integrated sleeve-in-drill system with irrigation channels (Adin® sCAIS system). The control group had neither drill handle nor sleeve-in-drill. The first pilot drill from each system was used, and drilling was performed for 10 seconds. The experiment was repeated 10 times for each group, and a blinded investigator measured the amount of irrigation solution in ml/s. A descriptive and bivariate analysis was performed.

**Results:**

The median volume of irrigant collected over 10 seconds was: Sleeve-in-sleeve group, 0.35 (IQR: 0.04) ml/s; sleeve-in-drill group, 0.07 (IQR: 0.04) ml/s; sleeve-in-drill system with channels group, 0.46 (IQR: 0.12) ml/s; and control group, 0.54 (IQR: 0.02) ml/s. The differences between groups were statistically significant (p=0.0001), except for the Adin sCAIS® system, which was not different from the control group (p=0.085).

**Conclusions:**

Within the limitations of the study, the volume of irrigation seems to be influenced by the design and sCAIS system used. Although all guides impeded irrigation, the Adin® sCAIS system facilitated irrigation the most.

## Introduction

Dental implants have emerged as a highly effective restorative treatment, offering a reliable solution for replacing missing teeth in partially and completely edentulous patients, with high survival rates (1 , 2). Nevertheless, the implant success rate may be reduced if implant positioning is not achieved through a prosthetically driven approach, due to possible biological or mechanical complications (3 , 4).

Recent advances in dental implantology include static computer-assisted implant surgery (sCAIS), which has significantly enhanced the accuracy and predictability of implant placement compared to the traditional freehand method (2 , 4). This system guides the drills and the implant to the planned position using a surgical guide. Although sCAIS allows accurate implant placement, the surgical guide limits vision of the surgical site, with a need for specific drilling kits, and the reduction of irrigation during the drilling protocol.

Osseointegration of dental implants depends on a dynamic biological process influenced by several factors, including the material, surface treatment and design of the implant, bone quality, implant loading protocol, surgical technique, and specifically, the heat generated during osteotomy (5). Controlling the heat generated by friction between the drill and the bone is crucial to minimize the risk of bone necrosis (6). In this regard, surgical guides can hamper drill irrigation of the drills and can increase the risk of overheating and bone necrosis.

Several guided drilling kit designs are available, such as systems with drill handle (also called sleeve-in-sleeve) or integrated sleeve-in-drill systems. All these sleeves, drills and components may inadvertently obstruct the coolant flow. Previous research has evaluated the cooling efficiency of different surgical guide sleeve designs (solid, window, porous) and irrigation strategies (5 , 6). However, only one in vitro study has quantified the volume of irrigation (7). Thus, the present in vitro study was designed to evaluate the volume of irrigation solution collected during implant drilling using different sCAIS systems, including a design with innovative irrigation technology in their drills, where channels inside each sleeve allow the coolant to flow through the sleeve during drilling.

## Material and Methods

Study design

An in vitro experimental study was conducted to measure the irrigation solution flow rate passing through the sleeve of three different surgical guides to the tip of the burr. The CRIS (Checklist for Reporting In vitro Studies) (8) guidelines were followed whenever possible throughout the study.

**Figure 1:**
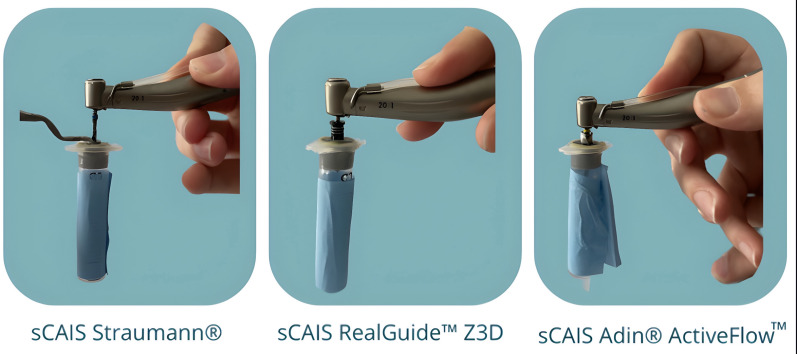
sCAIS systems tested.

The following sCAIS systems were tested (Figure 1):

1

1. A sleeve-in-sleeve system: The template has a metallic sleeve and a drill handle that reduces the diameter of the sleeve to the drill diameter (Straumann® Guided Surgery system).

2. An integrated sleeve-in-drill system. A template with a metallic sleeve, whose diameter matches the most proximal part of the drill. Hence, no drill handle is needed (RealGuideTM Z3D Guided Surgery system).

3. An integrated sleeve-in-drill system with irrigation channels. A template with a metallic sleeve, whose diameter matches the most proximal part of the drill. Hence, no drill handle is needed. Additionally, the wider part of the drill has a special design (ActiveFlow Irrigation Technology) with channels that facilitate flow of the irrigation solution through the sleeve while drilling (Adin® Guided Surgery system).

**Figure 2:**
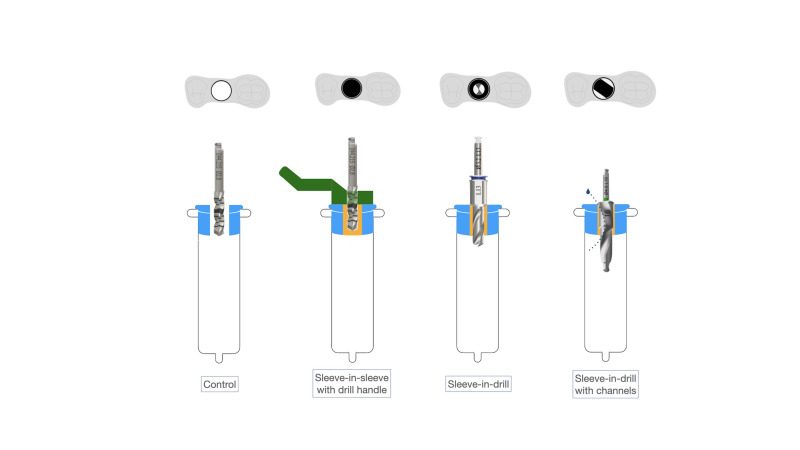
Study design: Occlusal buccal views. Control; Sleeve-in-sleeve with drill handle (Straumann® sCAIS system); Sleeve-in-drill (RealGuide™ Z3D sCAIS system); Sleeve-in-drill with irrigation channels (Adin® sCAIS system).

The same intervention was carried out in a control group using a template with a sleeve and a non-guided drill (Figure 2).

2

Sample size calculation

To calculate the sample size, use was made of the G* Power version 3.1.9.6 application (Universität Kiel, Germany), and flow rate (ml/min) was considered the primary outcome.

According to a recent in vitro study by Waltenberg et al. (7), a 4.2 ml/min (standard deviation, SD=1.2) flow rate is expected with an sCAIS sleeve-in-sleeve system with drill handle; a 5.8 ml/min (SD=1.2) flow rate with a surgical template with a lateral opening; and a 3.5 ml/min (SD=1.2) flow rate with surgical template without drill handle. If the study involves an allocation ratio of 1:1:1, a risk of 0.05 and a statistical power of 80%, a total of 7 repetitions were seen to be required for each group. To avoid possible deviations, 10 repetitions were made for each group.

Randomization, allocation concealment and blinding

In order to blind the researcher to the volume collected during drilling, the graded scale of the syringe was covered with opaque adhesive tape. The measurements were carried out by a second researcher, who was blinded to the system used.

Study sequence

The setup of the experimental phase involved the preparation of an in vitro clinical scenario simulating the drilling of an implant bed using sCAIS.

**Figure 3:**
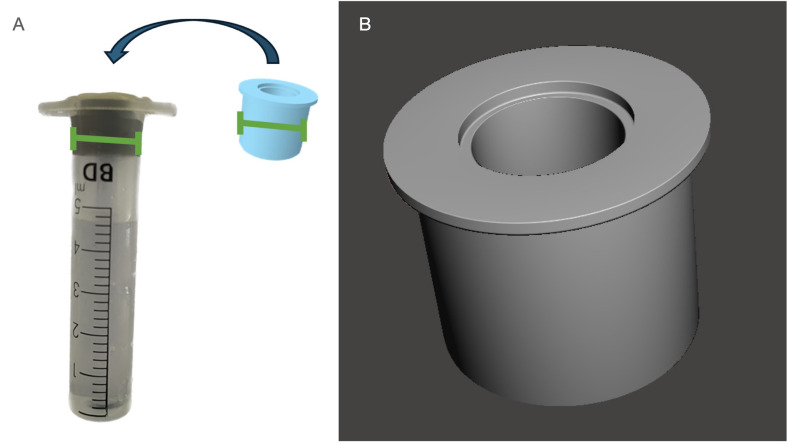
A: Customized 3D sleeve surgical guide fitting on the syringe. B: Final STL of the 3D sleeve surgical guide.

The measurement instrument was a modified Luer-lock® (BD DiscarditTM II, Huesca, Spain) syringe with the tip sealed with resin. The plunger of the syringe was removed and, in its place, a three-dimensional (3D) designed sleeve surgical guide, perfectly fitting the diameter of the syringe, was printed and placed on top (Figure 3).

3

For the design of the 3D sleeve surgical guide (Figure 3), Blueskyplan® software and Autodesk Meshmixer® were used. To begin the design process, the internal diameter of the syringe was calculated with a caliper (13mm), for application to the design of the external diameter of the ring by applying an offset calibration of 30µm (13.03mm). Using Blueskyplan®, two guide tubes were created. One presented the internal diameter of the guide ring, which was standardized at 6.50mm (since the manufacturer defines it at 6.30mm, leaving an offset of 200µm to allow for the different guides of the drills used), with a height of 10mm and an offset of 10mm. The other guide tube was made by adding 1mm to the internal diameter of the surgical rings, with a final diameter of 7.50mm (generating a settlement base between both tubes for the guide drills) and a height of 2mm. Once both guide tubes were parameterized, they were exported in standard tessellation language (STL) format and imported into the Meshmixer® software. Both objects were transformed with the following dimensions: Guide tube 1: 13.03mm external diameter and 10mm height. Guide tube 2: 14mm external diameter and 2mm height using the uniform scaling tool. Merging both 3D objects with the "merge" tool resulted in a ring adapted to the inside of the syringe and also the presence of lateral "wings" as a stop for the guide tube to settle on the syringe.

Once designed, calibration tests were performed with the Anycubic mono 4K printer. Anycubic grey resin with a wavelength of 365-405 nm was used, and the following calibration parameters were employed: 0.05mm layer height, 2.00 seconds exposure time, and a lifting speed of 120mm/min. Finally, post-processing was carried out using the Anycubic wash/cure machine, in which the excess material was first washed in a vat with 99% isopropyl alcohol for 15 minutes, then dried and post-polymerized for 20 minutes, obtaining a structurally clean and solid object.

Additionally, the perimeter of the surgical guide was sealed with resin to ensure no solution filtration, and the metallic sleeve was then placed and attached with resin to the surgical template.

In this study, the drilling sequence was performed using the first pilot drill (2.0 to 2.4mm diameter and 10 to 11.5mm implant length, depending on the sCAIS system used). The surgical system was set at the speed recommended by the manufacturer, and the irrigation rate was set at the highest level. Drilling was executed for 10 seconds, performing a repetitive movement with the drill going in and out, but always inside the sleeve. The same procedure was repeated 10 times for each group.

Outcome

A second researcher, blinded to the group tested, removed the tape from the syringe and measured the volume of the collected saline solution.

The volume of irrigation solution rate measured in milliliters per second (ml/s) was the main outcome variable of the study.

Statistical analysis

The normality of data distribution was assessed using the Shapiro-Wilks test and by examining the P-P plots. Normality distribution was not assumed for the main variable.

A descriptive analysis was conducted based on the median and interquartile range (IQR). Additionally, a bivariate analysis of the results was carried out using a Kruskal-Wallis test. Post hoc pairwise analysis was performed using the Dunn test with Bonferroni correction. The study methodology was reviewed by an independent statistician not involved in the study.

## Results

**Figure 4:**
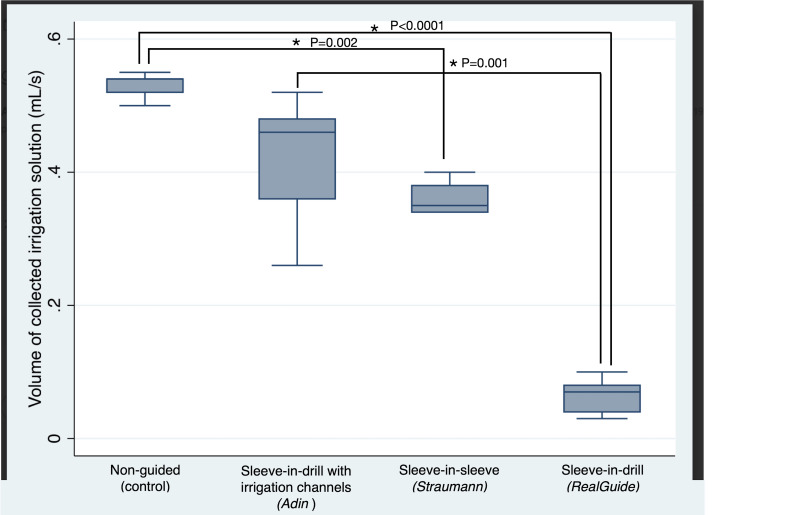
Boxplots of the collected irrigation solution volumes across all groups tested (*p&lt;0.05).

Ten drillings were performed for each of the groups. No protocol deviations were registered. The control group showed the highest median collected irrigation solution rate (0.54 (0.02) ml/s), followed by the sleeve-in-drill system with channels group (0.46 (0.12) ml/s) (the difference versus the control group being non-significant (p=0.085)), the sleeve-in-sleeve with drill handle system (0.35 (0.04) ml/s), and the sleeve-in-drill without drill handle system, which showed the lowest rate (0.07 (0.04) ml/s) (p=0.0001). The results obtained for all groups can be seen in Table 1 and Table 2 and are illustrated in Figure 4.

Table 1 Table 2

4

## Discussion

Overheating during implant placement using sCAIS systems is a critical concern in implant surgery, since excessive heat can negatively impact bone tissue and implant osseointegration. While surgical guides enhance accuracy by controlling the drilling trajectory and depth, they may limit cooling of the drills, generating higher temperatures in the bone. Without proper cooling irrigation, an important increase in bone temperature has been demonstrated, leading to bone necrosis or compromised implant osseointegration (5 - 7 , 9). Thus, understanding and managing heat generation appears to be essential for ensuring the long-term success of guided implant procedures.

The present study evaluated the flow rate of cooling solution passing through the surgical guide sleeve in three different sCAIS systems. The integrated sleeve-in-drill system with channels demonstrated the best irrigation rate, thanks to its novel drill shape. The presence of channels in these drills probably aided in directing the irrigation solution from the handpiece to the surgical site, optimizing irrigation during each drilling sequence.

Access of the irrigation solution to the surgical drill is a concern that has been addressed by several studies (9 - 13). Ahmet Orgev et al. (13) described a technique in which an irrigation channel is designed in the surgical guide that directly irrigates the drill after its pass through the sleeve. Hence, this technique ensures direct irrigation of the drill without being hindered by the sleeve.

When comparing the test groups versus the control group, a greater mean irrigation volume was consistently achieved in the control group. Although fully guided drilling templates significantly reduce the amount of cooling solution reaching the osteotomy site, Waltenberg et al. (7) demonstrated that the intraosseous temperature did not significantly decrease as a result. This suggests that while the design of the template may affect the flow rate, it does not necessarily lead to harmful temperature elevation during osteotomy. Indeed, other factors such as the irrigation system, bone density, drill shape, drilling speed, and drilling protocol have been proposed to play a significant role in the temperature rise during implant drilling (12 - 14).

Even though an exact threshold value for heat-induced osteonecrosis is not found in the literature, recent studies have correlated temperatures exceeding 47ºC for one minute with impaired osseointegration of the implant and its early failure (6 , 10 , 12 , 15). Nevertheless, further research should be carried out to establish a temperature threshold.

Nowadays, different types of surgical guides are available, including designs with side windows or perforations in the sleeve (9). In this regard, future studies comparing more sCAIS systems are needed to assess the impact of the design on both the drill and the implant bed temperature, as well as irrigation efficiency. Moreover, studies comparing the accuracy of different sCAIS systems or designs are also needed.

In recent years, keyless systems or sleeve-in-drill sCAIS systems as opposed to conventional drill-key (sleeve-in-sleeve) systems have been introduced seeking to reduce the system components and facilitate the surgical procedure (16). However, on comparing these two systems, sleeve-in-drill systems seem to significantly reduce the amount of irrigation that passes through the sleeve during implant placement. Indeed, in keyless systems, the most proximal part of the drill is specifically designed to fit the sleeve perfectly, preventing the creation of any space between the drill and the guide, and consequently reducing the space available for the solution to flow. In contrast, sleeve-in-sleeve systems have a key that perfectly fits the surgical guide sleeve to reduce its diameter to the drill diameter, but then the helicoid shape of the drill allows irrigation to flow through the key and the drill.

To enhance the irrigation of sleeve-in-drill systems, a novel design of sCAIS drills has been developed, featuring channels in the proximal part of the sleeve-in-drill mechanism. In our study, this sleeve-in-drill system with channels appears to provide the best irrigation rate. However, further studies are needed to confirm these preliminary findings. Additionally, it is important to carry on further studies assessing the accuracy of this novel system, since the non-circular shape of the drill, which does not fit tightly with the sleeve, could negatively impact implant placement accuracy. In fact, a simulation-based experimental study (17) comparing different sCAIS drilling systems found that sleeve-in-sleeve designs exhibited the lowest deviations in implant platform, apex and angulation. In contrast, systems without sleeves or using sleeve-in-drill mechanisms showed greater angulation deviations. This suggests that guide design and drill stabilization may influence implant placement accuracy.

According to Raabe et al. (18), the keyless system showed significantly smaller angular (3.1±1.7°) and apical (1.2±0.6mm) deviations than the drill-key system. Furthermore, a recent systematic review and meta-analysis (16) concluded that keyless systems allow for significantly better control of coronal implant deviation. Thus, both studies point to higher accuracy outcomes when using the keyless sleeve-in-drill system.

On investigating the effect of using surgical guides with or without sleeves upon the accuracy of implant placement, an in vitro study found that sleeveless guides provide greater accuracy compared to guides with sleeves (19). This appears mostly relevant in cases with limited space, since sleeves may interfere with proper fitting of the guide by contacting adjacent teeth. Moreover, although it has not yet been evaluated, friction between different types of materials (i.e., the metal of the drill against the metal of the sleeve or the resin of sleeveless systems) may also have an impact on heat generation by the drill.

Another important factor to consider is bone density. Type D1 bone is considered the most vulnerable to overheating (11). In fact, an in vitro study comparing open versus closed static CAIS reported that, regardless of the template used and with room temperature irrigation fluid (~21ºC), the 47ºC threshold was reached in the hard bone group (20). On the other hand, the same study confirmed the effectiveness of using chilled irrigation fluid (~5°C) to prevent heat generation exceeding 47°C in dense bone (20). Thus, the temperature of the irrigation solution could be another important factor to take into consideration during sCAIS drilling protocols.

Regarding drilling speeds, the most appropriate speed for preventing overheating remains the subject of discussion. While some implant systems recommend using between 800-1500 rpm with irrigation to avoid overheating, low-speed drilling without irrigation is also considered as an accepted method for preparing osteotomies (21). This approach may be especially beneficial in cases involving minimal simultaneous bone regeneration procedures, as it allows autologous bone to be harvested during drilling. On the other hand, increasing the drilling speed usually reduces the overall drilling time (22), thereby decreasing bone exposure to drilling and frictional heating. In this case, irrigation is essential to lower the temperature of the drill (10). Additionally, an in vitro study published by Salomó Coll et al. (14) observed that using external irrigation at higher drilling speeds (800 rpm) and omitting irrigation at lower speeds (50-100 rpm) were both effective strategies for preventing excessive heat generation, as the critical temperature threshold was not exceeded in either case.

The main limitation of this study lies in its in vitro design and the lack of bone tissue drilling, which prevents full reproduction of the clinical context. Additionally, the lack of drilling in synthetic, xenogeneic, or allogeneic bone models, as well as the absence of intraoperative temperature measurements during drilling, further limits the ability to directly correlate irrigation volumes with effective thermal control at the osteotomy site. Moreover, from a histological point of view, these parameters may be correlated with changes in bone structure, cell viability and implant osseointegration. These limitations should be taken into account when extrapolating the results to clinical scenarios.

Moreover, nowadays various types of surgical guides are available, as well as different sleeve materials (such as resins, zirconia or metals) and designs with side windows or perforations in the sleeve (9). Future research should therefore incorporate temperature monitoring during drilling in bone models and compare a broader range of sCAIS systems to evaluate the combined effect of guide design, sleeve configuration, and irrigation pattern on both cooling efficiency and drill/implant bed temperature (7). Thus, further investigation should focus on evaluating both accuracy and cooling effectiveness, aiming to correlate these two outcomes to identify the best design for an sCAIS system, thereby reducing the risk of overheating and implant failure.

## Conclusions

Overall, within the limitations of the study design, the flow rate of the irrigation solution passing through the sleeve of an sCAIS system is influenced by the design involved. Although all guides hamper irrigation to some extent, the sleeve-in-drill system with irrigation channels afforded the best irrigation rate during drilling.

## Figures and Tables

**Table 1:** Table Summary of the volume of collected irrigation solution.

System design	Median (IQR) ml/s
Sleeve-in-sleeve with drill handle (Straumann® sCAIS system)	0.35 (0.04)
Sleeve-in-drill with irrigation channels (Adin® sCAIS system)	0.46 (0.12)
Sleeve-in-drill (RealGuide™ Z3D sCAIS system)	0.07 (0.04)
Non-guided (control)	0.54 (0.02)
P-value	0.0001*

IQR: Interquartile range. AD: Absolute difference (|Mimic in vivo value-Ex vivo value|); MAD: Mean absolute difference; RE%: Relative error percentage (1) $\left(\frac{\left|\text{Mimic }\text{in vivo }\text{value}-\text{Ex vivo }\text{value}\right|}{\text{Ex vivo }\text{value}}\times 100\%\right)$ MRE%: Mean relative error percentage.

**Table 2:** Table Summary of the post hoc pairwise Dunn test analysis comparing the different groups. Each cell displays the median difference along with the 95% confidence interval (95% CI) in ml/s, followed by the p-value from the Dunn test.

	Non-guided (control)	Sleeve-in-drill with irrigation channels(Adin® sCAIS system)	Sleeve-in-drill(RealGuide™ Z3D sCAIS system)
Sleeve-in-drill with irrigation channels (Adin® sCAIS system)	0.09[95% CI: 0.03-0.18]0.085		
Sleeve-in-drill(RealGuide™ Z3D sCAIS system)	0.46[95% CI: 0.45-0.48]<0.0001*	0.42[95% CI: 0.29-0.43]0.001*	
Sleeve-in-sleeve with drill handle (Straumann® sCAIS system)	0.17[95% CI: 0.15-0.19]0.002*	0.1[95% CI: 0.02-0.13]0.747	0.3[95% CI: 0.27-0.32]0.057

2

## Data Availability

The datasets used and/or analyzed during the current study are available from the corresponding author.
